# Comparison of transanal endoscopic microsurgery with or without neoadjuvant therapy and standard total mesorectal excision in the treatment of clinical T2 low rectal cancer: a meta-analysis

**DOI:** 10.18632/oncotarget.22091

**Published:** 2017-10-26

**Authors:** Zheng-Shui Xu, Hua Cheng, Yuhong Xiao, Jia-Qing Cao, Fei Cheng, Wen-Ji Xu, Jia-Qi Ying, Jun Luo, Wei Xu

**Affiliations:** ^1^ Department of General Surgery, Xi’an N0.4 Hospital, 710000 Xi’an, Shanxi, China; ^2^ Department of General Surgery, The Second Affiliated Hospital of Nanchang University, 330006 Nanchang, Jiangxi, China; ^3^ The Second Clinical Medical College, Nanchang University, 330006 Nanchang, Jiangxi, China; ^4^ Department of Rehabilitation Medicine, The Second Affiliated Hospital of Nanchang University, 330006 Nanchang, Jiangxi, China

**Keywords:** rectal cancer, transanal endoscopic microsurgery, neoadjuvant therapy, total mesorectal excision, meta-analysis

## Abstract

Some clinical trials demonstrated local resection for clinical T1 rectal cancer was safe and effective. But for clinical T2 rectal cancer, the results were controversial. Neoadjuvant therapy (NT) is proven to reduce the opportunity of advanced rectal cancer recurrence in various researches. The objective of this Meta-Analysis was to evaluate the oncological outcomes of transanal endoscopic microsurgery (TEM) with or without NT comparing with conventional total mesorectal excision (TME) for the treatment of clinical T2 rectal cancer.To search for the relevant studies, an electronic search was done from the databases of Pubmed, Embase, and the Cochrane Library in this meta-analysis. We compared the effectiveness of transanal endoscopic microsurgery with or without NT and standard total mesorectal excision in the treatment of T2 Rectal Cancer. 1RCT and 3nRCTs including 121 TEM patients (TEM + NT: 59, TEM: 62) and 174 TME patients with T2 rectal cancer were retrieved. Compared with TME, there were no significant differences in the outcomes of local recurrence, overall recurrence, overall survival between TEM + NT group. However in compassion with TME, TEM without NT was associated with an increased local recurrence, overall recurrence, and a shorter overall survival, with individual ORs being 3.04 (95% Cl: 1.17–7.90; I^2^ = 0%), 5.67 (95% Cl: 1.58–20.38; I^2^ = 0%) and 0.12 (95% Cl: 0.02–0.65; I^2^ = 0%), respectively. Compared with TME, TEM after NT may be a feasible and safe organ preservative approach for patients with clinical T2 low rectal cancer. But for those without NT, TEM always seem be associated with worse oncological outcomes.

## INTRODUCTION

Rectal cancer remains one of the most common malignancies in the world, especially in the western countries and some developing countries [1, 2]. In the past twenty years, the five-year overall survival rate of rectal cancer was greatly increased, as a result of early detection and treatment was spread [1]. By 2009, early-stage rectal cancers (T1-T2) were nearly one quarter of all newly diagnosed ones in the UK [3]. Standard total mesorectal excision (TME) surgery, as another reason for the increased 5-year survival rate of rectal cancer, is an oncologically effective treatment for early-stage rectal cancers with extremely low rate of local and distant recurrence [4]. Unfortunately, TME-related complications are substantial, which include sexual and urinary dysfunction, troubling defecatory problems, decreased quality of life or a permanent stoma [5–7]. Some organ-preserving treatments were evaluated as the alternative approach of standard TME for the early-stage rectal cancers.

In 1983, Transanal endoscopic microsurgery (TEM) was firstly proposed for the treatment of large adenomas and clinical T1 rectal cancer [8]. But a major problem of TEM is the possibility of lymph node metastasis, which is the source for the local and remote recurrence, though its rate is very low in the early stage rectal cancer [9, 10]. The current evidence describes that TEM has gained general acceptance for the treatment of selected clinical T1 rectal cancer patients, because lots of reports demonstrate the same 5-year survival rate, similar recurrence rate and better functional results are followed with compared with standard TME resection [11–14]. But for clinical T2 rectal cancer, with higher rate of lymph node metastasis, the oncological outcomes and long-term survival are controversial. Neoadjuvant therapy (NT), compared with postoperative therapy, is proven to reduce the opportunity of advanced rectal cancer recurrence in a number of researches [15–17]. After all, NT maybe a suitable strategy for the probable lymph node metastasis leaved by TEM approach for the treatment of clinical T2 rectal cancer.

In the past 20 years, more and more practice experience were reported that TEM (with or without NT) maybe feasible and safety for treating clinical T2 rectal cancer [18–21]. Within these articles, some reports were compared the short-time outcomes and long-term survival between TEM (with or without NT) and standard TME, and there were many meta-analyses which assessed the effectiveness of TEM for stage (including T1 and T2) rectal cancer [18–21]. However, those results cannot be applied to T2 rectal cancer, because TEM as a surgical strategy for T1 rectal cancer has a consensus statement by NCCN Clinical Practice Guidelines in Rectal Cancer [22]. Those meta-analyses could not demonstrate the real pooled results in T2 rectal cancer with a too heavy weight of T1 rectal cancer. So we performed this meta-analysis to compare the effectiveness of transanal endoscopic microsurgery and total mesorectal excision in the treatment of T2 Rectal Cancer.

## MATERIALS AND METHODS

### Literature search

**Figure 1:**
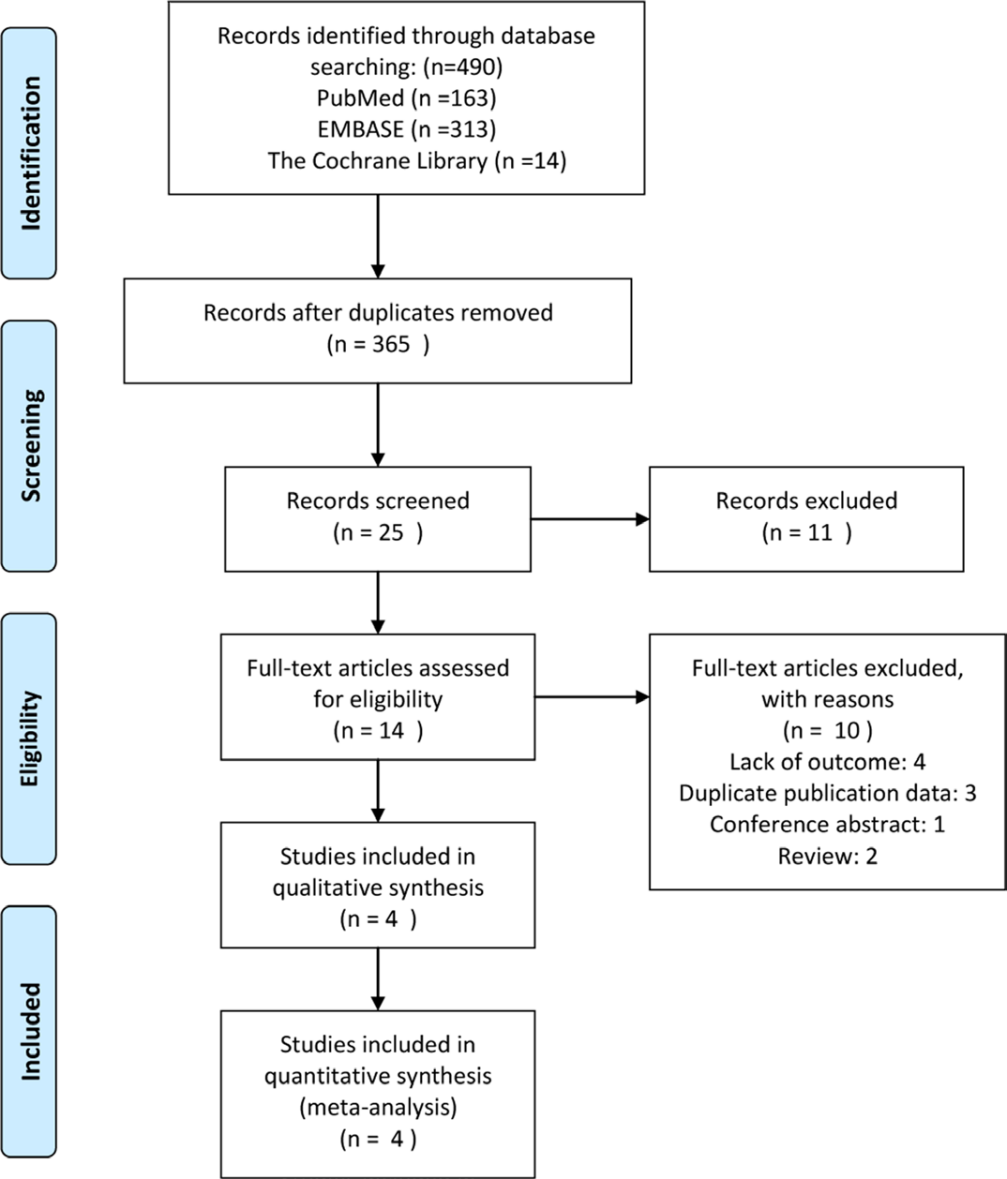
Diagram of study selection process

An electronic search was done from the databases of Pubmed, Embase, and the Cochrane Library to search for the relevant studies which were published from inception of these databases to march 19, 2016. No restrictions were entered for the search. The search strategy was performed using the following terms: rectal cancer, total mesorectal excision, transanal endoscopic microsurgery; Details of literature search were shown in appendix 1. Two investigators performed repeatedly the search strategy until no further relevant studies were retrieved; then two investigators assessed independently all the article using pre-designed eligibility forms; References of the above eligibility studies were also checked manually to add other potential eligibility studies. Any discrepancies were resolved by consensus. The scheme for this process was shown in Figure 1.

### Inclusion criteria and exclusion criteria

Eligibled studies should met the following criteria: (a) the diagnosis of T2 rectal cancer should be included pathological examination, clinical evaluation, colonoscopy and one or more imaging examination; (b) published studies comparing TEM and TME; (c) the sufficient data of our interesting. The excluded criteria was shown as follow: (a) the aim of trails were not T2 rectal cancer; (b) duplicate data or repeat analysis; (d) lack of sufficient data which we are interesting or cannot be calculated from the article data. Table 2 Pre-therapy protocol and follow-up adopted in included trials.

**Table 1:** Characteristics of included studies in current meta-analysis

Author	Year	Country	study design/quality*	Age(years)	Gender (M/F)	Neoadjuvant therapy	Follow-up (months)	TNM stage	Histological grade
Allaix	2012	Italy	Retrospective analysis of Prospective database/6	72(38–91) 65 (34 –90)	16:2516:19	A part of patients	70 (36–140)	T2N0M0	NA
Chen	2013	China	Prospective Studies/6	68.8 ± 5.3 66.2 ± 7.7	14:1617:13	No patient	18 ± 2.6 17.5 ± 2.2	T2N0M0	G1-2
Lee	2003	South Korea	Retrospective/8	61.1 ± 11.2 57.7 ± 11.8	37:3750:50	No patient	31 ± 17.234.6 ± 19.4	T2N0M0	G1-2
Lezoche	2012	Italy	Randomized, controlled trial/4	66 (58–70) 66 (60–69)	30:2034:16	All patients	115.2 (102–133.2)115.2 (88.8–142.8)	T2N0M0	G1-2

^*^ Quality assessment was done using the Newcastle–Ottawa Scale (range 0–9) for non-randomised studies and using a Jadad score (range 0–5) for randomized, controlled trials. NA: No applicable.

**Table 2:** Pre-therapy protocol and follow-up adopted in included trials

Trial	TEM group	TME group
Allaix 2012	Neoadjuvant therapy: radiotherapy of 45 Gy for 6–8 weeks for all TEM patients. Two cases (18 %) of all neoadjuvant treatment with local tumor progression (both patients underwent open surgery) were observed, in this condition we supposed that two cases had the worst results that both cases were classified as “event group”. Because of one patient lost to follow-up 11 patients, 32 patients undergone TEM+NT, TEM only, respectively.	No neoadjuvant treatment. Because of 2 patient lost to follow-up, That 33 patients undergone TME were inclued in our study.
Chen 2013	No neoadjuvant treatment. 8 patients undergone TEM. No patient lost to follow-up.	No neoadjuvant treatment. 8 patientsundergone TME. No patient lost to follow-up.
Lee 2003	No neoadjuvant treatment. 22 patients undergone TEM. No patient lost to follow-up.	No neoadjuvant treatment. 83 patientsundergone TME. No patient lost to follow-up.
Lezoche 2012	Neoadjuvant therapy: long course 3D four-field chemoradiotherapy in prone position, with bladder prep and use of IV contrast Total dose 5,040 cGy in 28 fractions over 5 weeks with infusion of 5-fluorouracil 200 mg/m^2^ per day during radiotherapy. 50 patients undergone TME. No patient lost to follow-up.	The same neoadjuvant treatment for all patient. 50 patientsundergone TME. No patient lost to follow-up.

### Data collection and quality assessment

The data of our interest were extracted independently; the results were compared and checked by another co-author. From each study the following information were collected: first author, year of publication, country of origin, study design, age, gender, total number of cases, with or without NT, the outcome (local recurrence, overall recurrence, overall survival, postoperative complications) and follow-up. The quality assessment of non-randomized controlled clinical trials (nRCTs) or randomized controlled trials(RCTs) was done using the Newcastle–Ottawa Scale (range 0–9) [23] or a Jadad score (range 0–5) [24], respectively.

### Statistical analysis

We conducted this meta-analysis according to the recommendations of the Preferred Reporting Items for Systematic Reviews and Meta-Analysis (PRISMA) Statement. [25] Heterogeneity among the included studies was evaluated using the I^2^ values. An I^2^ value of is equal or lesser than 50% showed no significant heterogeneity, and the fixed-effects model was used. Otherwise, a random-effects model was reported, and it represented substantial levels of heterogeneity. Because of all pooled outcomes which were less than 10 included trials, publication bias would not be evaluated. All data were analyzed using the Review Manager Version 5.2 (The Cochrane Collaboration, Oxford).

## RESULTS

### Study characteristics

The initial database search identified 490 publications. Through removing the duplicates by electron and handed-scan, 365 publications were left. After reviewing the title and abstract of all the 365 publications, we identified 14 potential eligible studies. Finally 6 studies was left, however 3 studies [26–28] reported the similar outcomes at different follow-up point. Eventually, 1RCT and 3nRCTs including 121 TEM patients (TEM + NT: 59, TEM: 62) and 174 TME patients with T2 rectal cancer were retrieved, which published between 2003 and 2013 [28–31]. In the Allaix’s study, two cases (18%) of all patients with local tumor progression in neoadjuvant treatment group - both patients underwent open surgery were observed, in this condition we supposed that two cases had the worst results that both cases were classified as “event group” [30]. The characteristics of all including studies were summarized in Table 1, and the pre-therapy protocol and follow-up adopted in included trials were presented in Table 2.

### Oncological outcomes

#### TEM + NT vs TME

**Figure 2:**
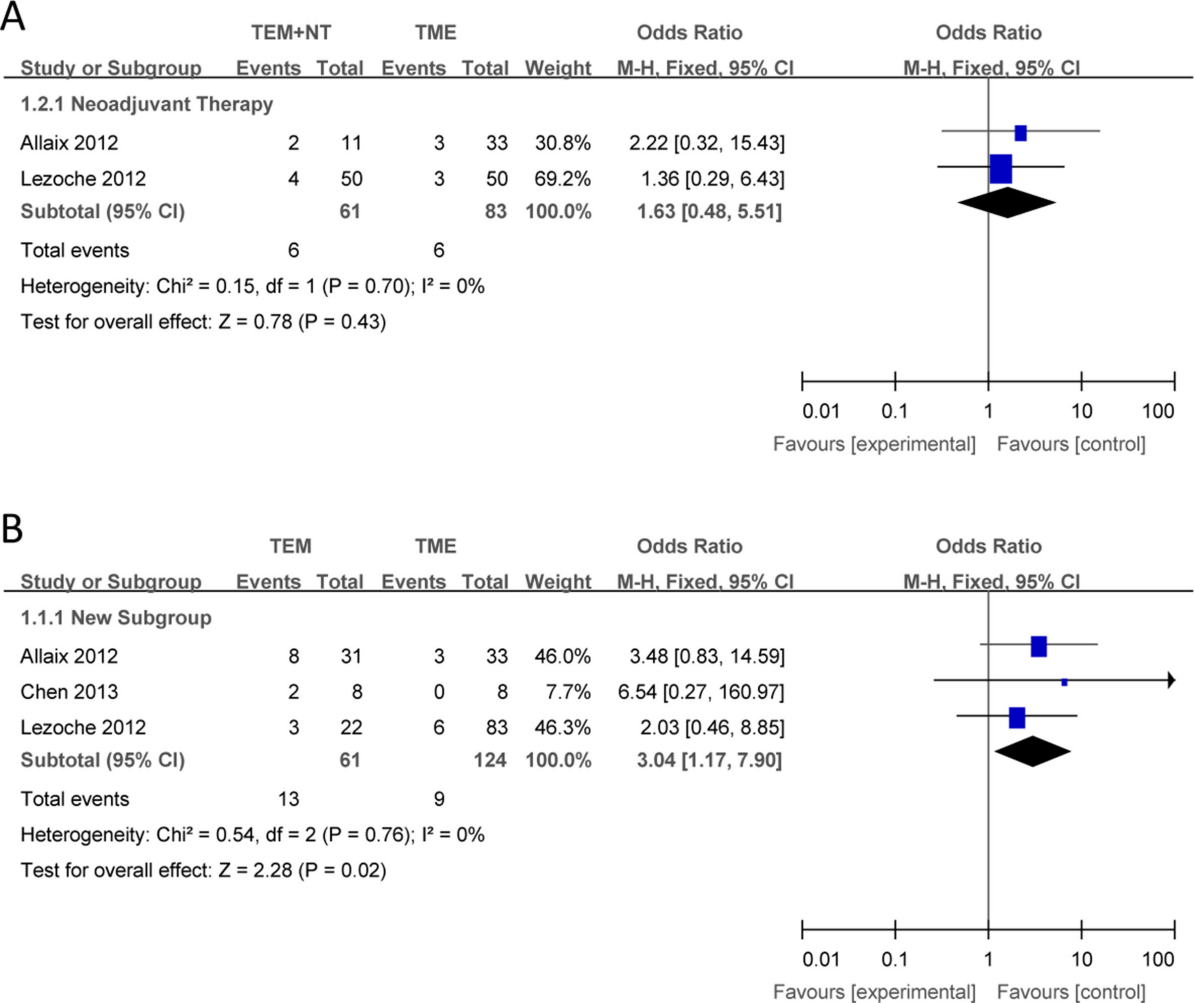
Forest plots of local recurrence, TEM + NT VS TME (**A**), TEM only VS TME (**B**). TEM = transanal endoscopic microsurgery, TME = total mesorectal excision, NT = neoadjuvant therapy, CI = confidence intervals, MH = Mantel–Haenszel.

**Figure 3:**
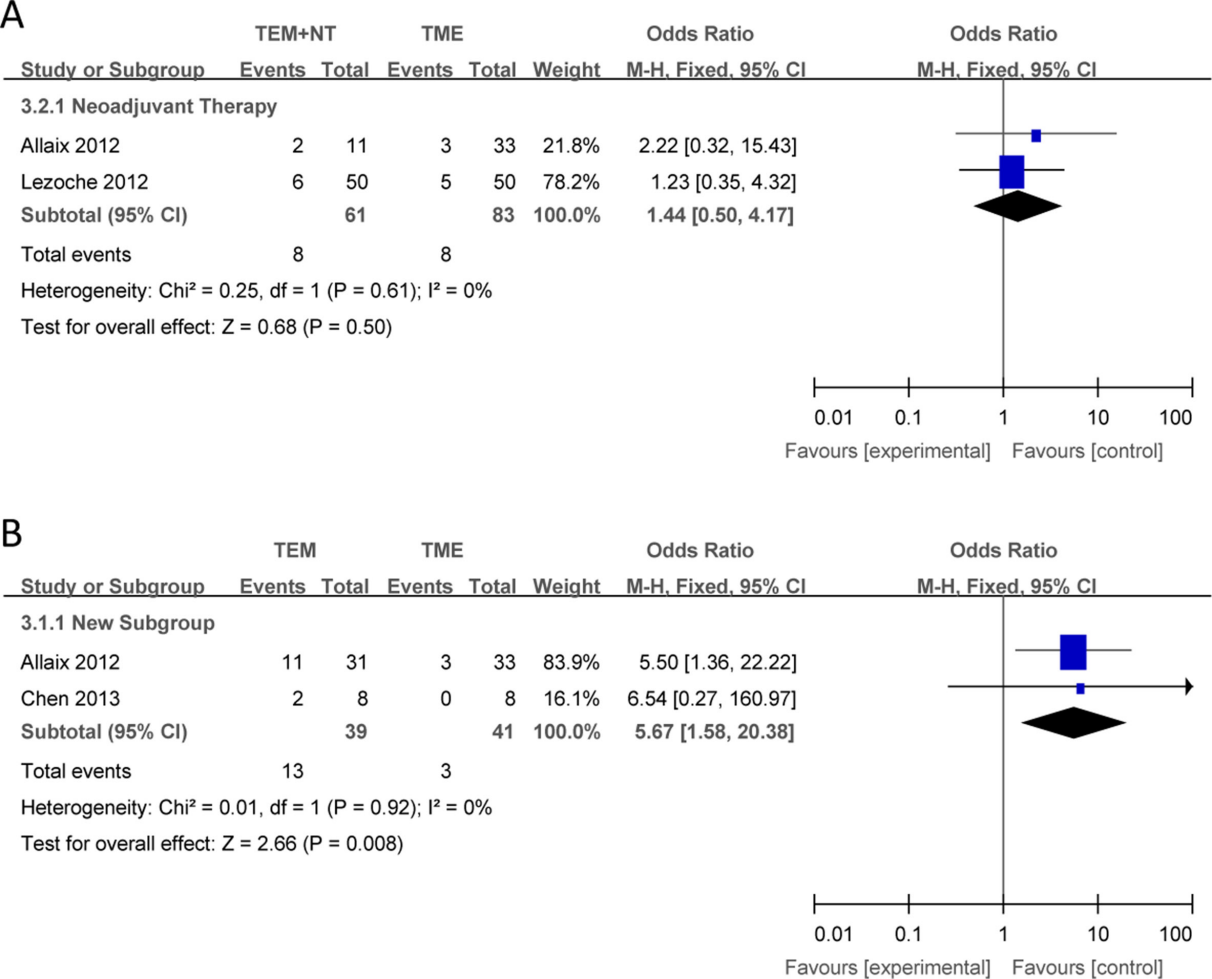
Forest plots of local recurrence, TEM + NT VS TME (**A**), TEM only VS TME (**B**). TEM = transanal endoscopic microsurgery, TME = total mesorectal excision, NT = neoadjuvant therapy, CI = confidence intervals, MH = Mantel–Haenszel.

**Figure 4:**
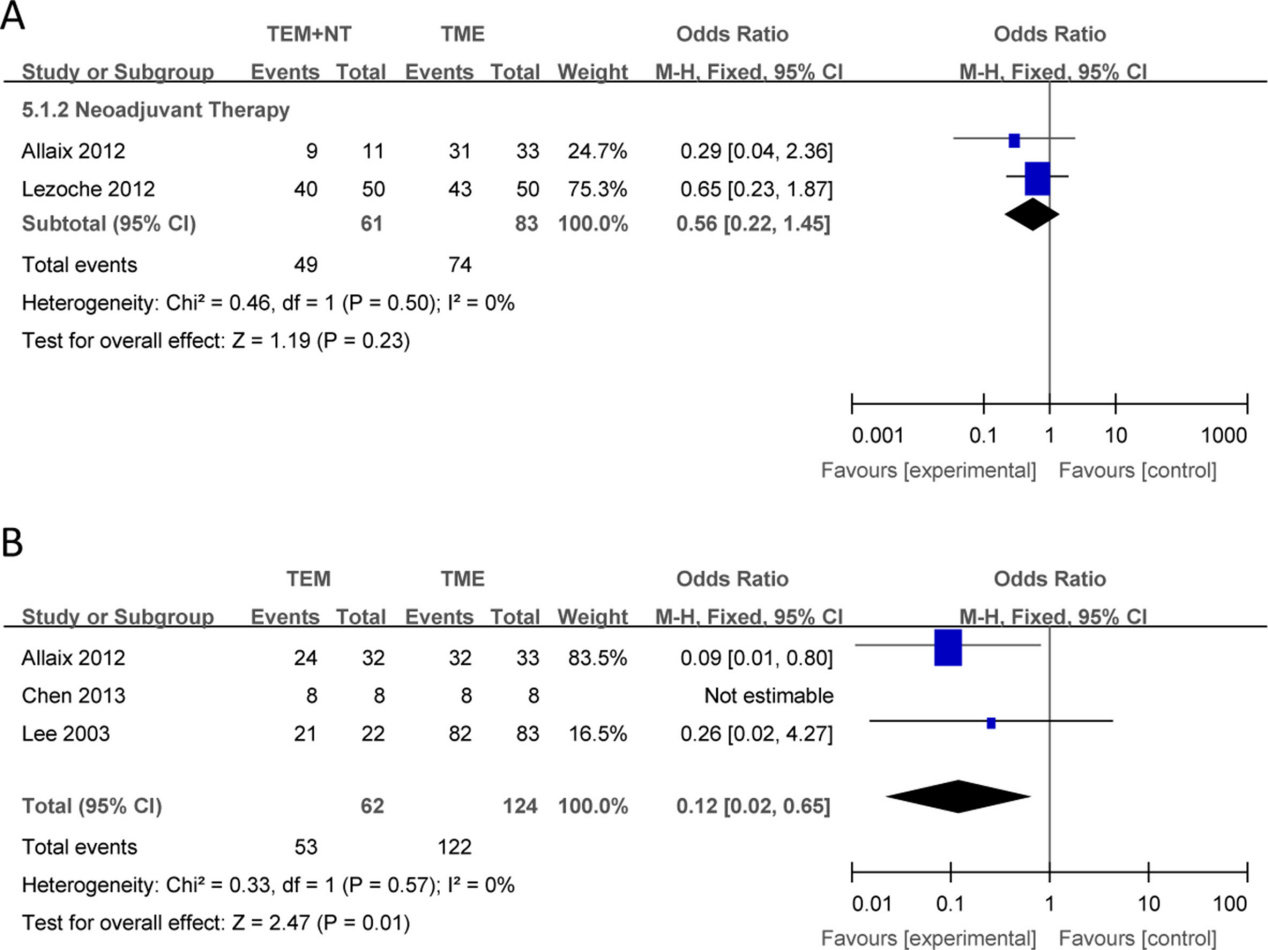
Forest plots of overall survival, TEM +NT VS TME (**A**), TEM only VS TME (**B**). TEM = transanal endoscopic microsurgery, TME = total mesorectal excision, NT = neoadjuvant therapy, CI = confidence intervals, MH = Mantel–Haenszel.

Compared with TME, there were no significant differences in the outcomes of local recurrence (Figure 2A), overall recurrence (Figure 3A), overall survival (Figure 4A) between TEM + NT group.

#### TEM only vs TME

Compared with TME, TEM only was associated with a increased local recurrence (Figure 2B), overall recurrence (Figure 3B), and a shorter overall survival (Figure 4B), with individual ORs being 3.04 (95% Cl: 1.17–7.90; I^2^ = 0%), 5.67 (95% Cl: 1.58–20.38; I^2^ = 0%) and 0.12 (95% Cl: 0.02–0.65; I^2^ = 0%), respectively.

## DISCUSSION

Low anterior resection (LAR) or abdominoperineal resection (APR) with total mesorectal excision is widespread accepted as the gold standard procedure in early stage rectal cancer, with the same oncological results between the open and the laparoscopic approach [32–34]. Laparoscopic surgery has proven to have better short-term clinical outcomes than open approach, which is also burdened by high rate of morbidity and mortality. The rate of mortality was 2% to 6% after radical rectal surgery, and this rate could be higher in elderly patients [35, 36]; the major complications were urinary and sexual dysfunction, long-term functional bowel disturbance, and anastomotic leakage [37–39]. With temporary or permanent stoma, patients may suffer significant psychological problems, which will lead to worsening of the quality of life (QoL) [40, 41]. For early stage rectal cancer, oncological outcomes should be the primary aim, and secondly preserving function and QoL should pay more attention on.

The poor QoL and high rate of morbidity drive surgeons to reconsider the role of local therapy for early stage rectal cancer, which could preserve integrity of anal sphincter and pelvic autonomic nerve to maintain the best QoL. TEM, as a kind of local excision, was firstly introduced for the treatment of large rectal polyps and early rectal cancer in 1983 [42]. As compared with traditional local resection approach, TEM achieves a better vision of operation area, which allows a very accurate and full-thickness excision of the tumor and mesorectum down to the ‘holy plane’ with an about 1cm surrounding tumor-free margin. A R0 resection margin of the specimen is significant importance for the rate of local recurrence.

Currently, TEM for rectal magligant tumor is just accepted for clinical T1 adenocarcinomas with favorable prognostic characteristics. In these patients, the rate of local recurrence and overall survival is similar compared with conventional radical TME surgery [43–45]. But for clinical T2 rectal cancer, with about 20% lymph node metastasis rate, the oncological outcomes and long-term survival of TEM are controversial [46]. Following the development of NT in rectal cancer, it improves the local control of rectal cancer. NT can lead to a complete clinical response (CCR) in 10%–30% of rectal patient [47]. This result brings about a new “wait and see” strategy. However smith et al [48] found that the residual mucosal abnormalities which were less than 3 cm had significantly association with ypT0-1 after neoadjuvant therapy for rectal cancer. And a CCR did not mean a complete pathological response (CPR) in some cases. So TEM + NT could be commended as an ontologically adequate treatment. But there are a very limited number of clinical trials comparing TEM with or without neoadjuvant therapy and radical surgery for clinical T2 rectal cancer. Our present research analyzed 4 studies, including 1 RCT + 3nRCT, which are focused on different short-term and long-term outcomes. Our primary endpoint is the local recurrence, overall recurrence and overall survival, which are paramount points of oncological outcomes. As the results described in our meta-analysis, for patients with clinical T2 low rectal cancer after NT, the local recurrence, overall recurrence and overall survival is similar with TME surgery; unfortunately, for clinical T2 patients without NT, the results of local recurrence rate, overall recurrence and overall survival are significantly worse than radical surgery. NT is the key point for this favorable outcome, because of it could reduce the local infiltration depth of cancer and the possibility of mesorectal lymph nodes metastasis [15–17].

Besides these, we must emphasize the advantage of TEM in terms of QoL. Although a nerve sparing technique is applied to laparoscopic TME, the anterior resection syndrome, including urinary dysfunctions, sexual, and variable defecation, still occurs with the rate of 50%-90% [49–52]. Obviously, TEM thoroughly win in this term [53, 54]. Moreover two studies reported that there are no significant differences in QoL between TEM and TEM after NT for locally advanced rectal cancer [54–55]. Many authors recommended that a temporary or definitive stoma should be applied to low anterior resection, it goes without saying that it would increase the prevalence of depression and worse the quality of life and, especially in some cultural contexts [41, 56].

Many studies proved that TEM with or without NT have a better short-term outcomes compared with TME, including blood loss, transfusions, operating time, need for analgesia and hospital stay [27, 43, 57]. Compared with TME, there are lower life-threatening complication occurred after TEM with or without NT, this advantage would be obvious in the patient with American Society of Anesthesiologists’ (ASA) class III or IV [27, 57].

However, every nutshell has a concave and convex side. Firstly, chemoradiotherapy-related toxicity is a concern in the patient with early stage rectal cancer who should not have to receive neoadjuvant therapy before TME, mortality incident is the most fearful; although some studies reported that mortality incident on chemoradiotherapy is less than 1% [57, 58]. Secondly, treatment-related toxicities after TEM+NT can be neglected, including anorectal pain, proctitis, diarrhoea, suture dehiscence and persistent confusion et al. [58, 59]. Thirdly, some patients who treated with TEM + NT may still need TME; these patients were overtreatment with additional morbidity of NT and TEM [58]. Because of the fibrotic scar in the rectal wall and the local inflammation after TEM, the pelvic dissection and a low colorectal or a coloanal anastomosis would be more difficult [43]. Beiside these, NT would increase the rate of anastomositis, marginal ulcer, anastomotic fistula et al. [43, 57–60].

What are the future problems in the development of this procedure? It is not completely accurate for the preoperative staging of the tumors, compared with the definitive histological stage. Even though, EUS was considered as the most accurate preoperative diagnostic tool for the tumor invasion of the rectal wall and high quality of MRI for the lymph node condition. What are next steps in the development of this procedure? The next study should be refined. Firstly, it is important that ypT2 rectal cancer should be divide into a group, because it is the most ambiguous whether TEM + NT is applicable to ypT2 rectal cancer. Secondly, it should be clear whether the diameter, the different pathologic types and grades of T2 rectal cancer would make different outcomes. Finally, whether and how would TEM + NT be applicant to clinical T3 rectal cancer?

There were some limitations to our study. First, there are an insufficient number of research studies in comparsion of transanal endoscopic microsurgery with or without NT and standard total mesorectalexcision in thetreatment of clinical T2 low rectal cancer, and only 1RCT and 3nRCTs with nearly 300 patients were included in this meta-analysis. Second, because of insufficient data of short-term outcomes, they cannot be analyzed in this meta-analysis. Moreover, these 4 including studies were published in English, so publication bias cannot be excluded. Thus, we reckoned that more attention should been paid to TEM with NT in the treatment of clinical T2 low rectal cancer, and this is one reason why we written this article.

## CONCLUSIONS

Compared with TME, TEM may be a feasible and safe organ preservative approach for patients with clinical T2 low rectal cancer after NT. But for those without NT, TEM always seem be associated with worse oncological outcomes. Anyhow it is good news for the patients with T2 low rectal cancer refusing abdominal surgery or unfit for TME because of severe comorbidities.
